# Biocompatibility of *Bletilla striata* Microspheres as a Novel Embolic Agent

**DOI:** 10.1155/2015/840896

**Published:** 2015-09-09

**Authors:** ShiHua Luo, SongLin Song, ChuanSheng Zheng, Yong Wang, XiangWen Xia, Bin Liang, GanSheng Feng

**Affiliations:** Department of Radiology, Union Hospital, Tongji Medical College, Huazhong University of Science and Technology, 1277 Jiefang Road, Wuhan 430022, China

## Abstract

We have prepared Chinese traditional herb *Bletilla striata* into microspheres as a novel embolic agent for decades. The aim of this study was to evaluate the biocompatibility of *Bletilla striata* microspheres (BSMs). After a thermal test of BSMs in vitro, the cell biocompatibility of BSMs was investigated in mouse fibroblasts and human umbilical vein endothelial cells using the methyl tetrazolium (MTT) assay. In addition, blood biocompatibility was evaluated. In vivo intramuscular implantation and renal artery embolization in rabbits with BSMs were used to examine the inflammatory response. The experimental rabbits did not develop any fever symptoms after injection of BSMs, and BSMs exhibited no cytotoxicity in cultured mouse fibroblasts and human umbilical vein endothelial cells. Additionally, BSMs exhibited high compatibility with red blood cells and no hemolysis activity. Intramuscular implantation with BSMs resulted in a gradually lessened mild inflammatory reaction that disappeared after eight weeks. The occlusion of small renal vessels was associated with a mild perivascular inflammatory reaction without significant renal and liver function damage. In conclusion, we believe that BSMs exhibit high biocompatibility and are a promising embolic agent.

## 1. Introduction

In the last decade, various embolic agents have been developed to further improve the safety and efficacy of embolization procedures. In particular, the development or refinement of spherical embolic particles has remarkably increased the spectrum of interventional radiology. Spherically shaped particles were introduced into the armamentarium of embolization techniques to overcome the disadvantages of irregularly shaped particles, such as the promotion of catheter clogging, incomplete occlusion of the target vessels, and unpredictable behavior [1]. Conversely, spherical embolic particles show a more uniform distribution in target vessels, and the occlusion level can be predicted according to the particle size chosen; furthermore, fewer incidences of collateral circulation have developed after embolization [2].

The primary component of* Bletilla striata* (BS) polysaccharide (BSP) extracted from the traditional Chinese medicine BS is the polysaccharide macromolecule glucomannan [3], which is generated from the polymerization of four mannoses and one glucose and produces well-known anti-inflammatory and antineoplastic effects [4, 5]. In the last two decades, our research group [6, 7] has shown that the characteristics of BS have met the requirements as a potential peripheral embolic agent. Presently, we further optimize the extraction of BS and prepare* Bletilla striata* microspheres (BSMs) of different sizes. The biocompatibility of BSMs was observed both in vitro and in vivo, the findings of which may help us to examine the feasibility of applying BSMs as potential embolic agents.

## 2. Materials and Methods

### 2.1. Preparation of BSMs

*Bletilla striata* polysaccharide was prepared by ethanol precipitation following 60°C water extraction, deproteinization using the Sevage method, petroleum ether (Sinopharm Chemical Reagent Co., Beijing, China) defatting, and activated carbon (Sinopharm Chemical Reagent Co., Beijing, China) bleaching. The polysaccharide was further isolated by ion-exchange chromatography on a DE-52 column (Whatman Co., Kent, UK) and gel filtration on a Sephadex G-100 column (Whatman Co., Kent, UK), and purified BS polysaccharide was obtained. BSMs were prepared according to the modified method of emulsion-condensation-chemical cross-linking [7]. The microspheres were sized by passing them through sieves with different apertures. Next, they were placed into bottles based on sphere size and then packaged and sterilized with ^60^Co-*γ* exposure for disposal.

### 2.2. Characteristics of BSMs

**Figure 1:**
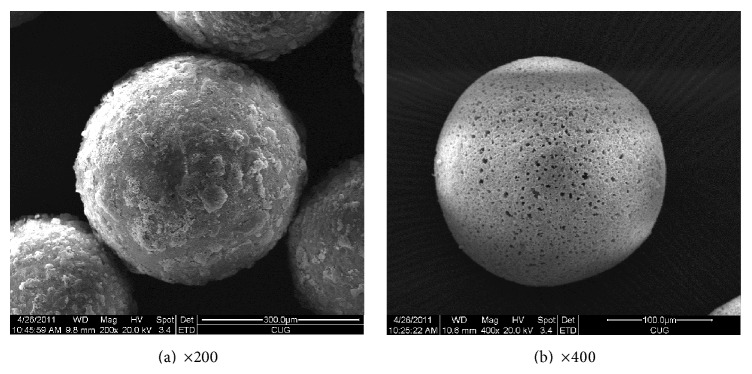
Photograph of particle by electron microscopy. BSMs were regular and uniform in a series of sizes without aggregation. Small holes were noted on the surface of the microspheres.

Scan-electromicroscope (FEI Co., Eindhoven, Netherland) showed that the BSMs were regular and uniform in size and without aggregation. Small holes were noted on the surface of microspheres (Figure 1). The microspheres were sized by passing them through sieves with different apertures to 50–100 *μ*m, 100–200 *μ*m, 200–300 *μ*m, 300–400 *μ*m, 400–500 *μ*m, 500–700 *μ*m, 700–900 *μ*m, 900–1200 *μ*m, and 1200–1400 *μ*m in diameter, and then they were placed into bottles based on the sphere size, packaging, and sterilization. BSMs dispersed well in normal saline and Omnipaque (Omnipaque 350; GE Healthcare, Shanghai, China). The settling times in these 2 solutions were long enough for syringe drawing. The microspheres went well through 5F catheter in both normal saline and Omnipaque solution. There was no aggregating and deformation. Microsphere size less than 400 *µ*m could pass through 3F SP microcatheter (Terumo Corporation, Tokyo, Japan) and was not deforming and aggregating. For microspheres with diameter larger than 400 *µ*m, it was difficult to pass through a 3F SP microcatheter. They would aggregate, swell, and occlude the microcatheter. The characteristics of BSMs in vitro met the requirements for interventional approaches that appeared in other manuscripts.

### 2.3. Animal Experiment Protocol

Our Animal Care and Use Committee approved all of the experiments (SCXK2011-0011), and eighty-eight animals were used in this study. New Zealand rabbits, both male and female, weighing 2.0–2.5 kg, were obtained from the Laboratory Animal Center of Tongji Medical College, Huazhong University of Science and Technology. After the surgery, analgesic promethazine (2.5 mg/kg, Beijing Double-Crane Pharmaceutical Co., Ltd, China) was injected intramuscularly for pain control. All the animals were returned to their cages after surgery, kept warm, and monitored in the animal laboratory twice in one day. Humane endpoints were used and animals were euthanized prior to the end of our experiments according to AVMA Guidelines on Euthanasia, 2007, American Veterinary Medical Association.

### 2.4. Thermal Test of BSMs

A thermal test was conducted by the thermal inspection method specified in Chinese Pharmacopoeia (2010 version). Warm BSMs in physiological saline solution (PSS) at approximately 38°C were injected into ear veins of the selected three New Zealand rabbits within 15 min after taking their baseline temperature; their temperatures were then taken 6 times in 30-min intervals. Subsequently, the temperature increment of the experimental rabbits was obtained by subtracting the normal temperature from the highest temperature. Another five rabbits were used for the above experiments in the same test method again if the temperature of one experimental rabbit rose by 0.6°C or higher or if the temperatures of all experimental rabbits rose by 1.3°C or higher.

*Result Determination.* The thermal tests of the rabbit samples were considered in accordance with regulation when the temperature increment of each experimental rabbit was lower than 0.6°C, the total increment of the three rabbits was lower than 1.3°C in the first test, and the total temperature increment of eight experimental rabbits was 3.5°C or lower in the first and second tests. The thermal tests of the rabbit samples were considered in discordance with regulation when the temperature of more than one rabbit rose by 0.6°C or higher in the three rabbits for the first test, the temperature of more than one rabbit rose by 0.6°C in the five rabbits for the second test, or the total increment of the eight rabbits was higher than 3.5°C in the first and second tests. The negative temperature increment values were considered 0°C.

### 2.5. Cytotoxicity of BSMs

Five milligrams of sterilized BSMs was diluted in 1 mL of culture medium, and a 30-*µ*L BSM suspension was added per well of a 96-well plate. Mouse fibroblasts and human umbilical vein endothelial cells in the log growth phase were prepared for cytotoxicity experiments after trypsin digestion. Two hundred microliters of the cell suspension at a density of 1–10 × 10^4^ was seeded into 96-well plates and mixed with BSMs. Three parallel wells were set for each sample. The plates were placed in a humidity culture chamber at 37°C and 5% CO_2_. Cell viability was observed using the methyl tetrazolium (MTT) method 1 h, 4 h, 24 h, and 72 h after incubation. Blank medium without BSMs was set as the negative control. Fifty microliters of fresh MTT solution (5 mg/mL) was added to each well and incubated for 4 hours. Next, 200 *µ*L of dimethyl sulfoxide (DMSO) was added to each well and shaken throughout the evening using a flat shaking table. Finally, the optical density (OD) was determined using a microplate reader (the detection wave length is 570 nm). Cells treated with blank medium without BSMs were set as the control group. The inhibition ratio was calculated according to the following formula: inhibition ratio = (1 − OD in the experimental group/OD in the control group) × 100%.

### 2.6. Blood Compatibility of BSMs

Blood was obtained from one New Zealand rabbit by cardiac puncture before euthanasia using intravenous injections of 30 mg/kg of sodium pentobarbital. Defibrinated blood was prepared after filtering the whole blood with sterile absorbent gauze. One hundred fifty milligrams of BSMs, which was diluted with 10 mL of physiological saline, was added to 0.1 mL of defibrinated rabbit blood and stored at 37°C for 24 h. Determination standard was solution layering without hemolysis. If there is no layering of the solution by gross observation, then it indicated occurrence of hemolysis, lastly observed erythrocyte state with light microscope after Wrights staining of erythrocyte at the vascular bottom.

Five milliliters of whole blood (heparin anticoagulation) and 5 mL of 2% erythrocyte-physiological saline suspension were obtained using the method mentioned above and then added to the test tubes. Finally 100 mg of BSMs was added to the test tubes separately and mixed evenly, and the samples were taken after storing the test tubes at 4°C in the fridge for 6 h and 24 h, respectively, to observe erythrocyte morphology with a light microscope after Wrights staining.

### 2.7. Intramuscular Implantation of BSMs

Twelve New Zealand rabbits were used in the present intramuscular implantation study. Under anesthesia and sterile conditions, two implanted points were selected at the same distance from the spine, and a pocket was made in the gluteal muscle at a depth of 1 cm. Polyvinyl alcohols (PVAs, COOK Co., Bloomington, USA) and BSMs of 1 mg were implanted on each side. Finally, the fascia, muscle, and skin layer were sutured. One week, 4 weeks, and 8 weeks after implantation, 4 rabbits were sacrificed at each time point, and the implant sites were excised and removed with the surrounding muscle tissue. The samples were fixed in 10% formaldehyde, embedded in paraffin, sectioned, and stained using the hematoxylin-eosin (HE) method.

### 2.8. Intra-Arterial Embolization of BSMs

Seventy-two New Zealand rabbits, regardless of gender, were subjected to embolization, after anesthesia, on the right renal artery with BSMs (300 *µ*m) or PVA with the same size as the control group. There were 36 rabbits each in the experimental group and control group, with four rabbits for each of nine periods to observe liver and kidney functions. Renal tissue slices were embedded in paraffin and subjected to vascular fiber Masson staining. Then, renal target vascular wall changes and the inflammatory response of surrounding cells were observed after embolization.

Angiographic procedures were performed using a Siemens C-Arm Power Mobil unit (Angiostar Plus, Siemens Medical Solutions, München, Germany). After fasting the rabbits for 12 hours, all of the experimental rabbits were subjected to anesthesia and were fixed on a wooden plate in the supine position. For the procedure, the right common femoral artery was surgically cut, a 4-F catheter sheath (Terumo, Tokyo, Japan) was introduced, and a 4-Fr Cobra visceral catheter (Terumo) was used to select the abdominal aorta through which digital subtraction angiography (DSA) was performed. The trunk of the renal artery was selected using a 4-Fr Cobra-type catheter, and renal arteriography was performed by manual injection of 2 mL of Omnipaque, diluted to 50%. The renal artery was embolized immediately after the administration of the mixture of embolic agents. At each time point after embolization, angiography and pathological examination of kidneys as well as liver and renal function tests were performed. All of the animals were returned to their cages after surgery, kept warm, and monitored in the animal laboratory until they recovered from anesthesia. They were monitored after the operation and given analgesics if they showed any sign of physical distress. None of the animals died during that phase of the protocol.

### 2.9. Statistical Analysis

SPSS 17.0 statistical software was adopted for analyses, the measurement data are expressed as the mean ± SD, and the difference was regarded as statistically significant with a *P* value of less than 0.05. Nonparametric Wilcoxon signed-rank test was applied for paired data (intragroup comparison). For the comparison of unpaired data (intergroup comparison: after embolization with PVA versus BSM), the nonparametric Mann-Whitney *U* test was applied.

## 3. Results

### 3.1. Result of BSM Thermal Tests

The rectal temperatures of the three rabbits were 38.5°C, 38.6°C, and 38.5°C before BSM injection and slightly increased after injection. The temperature increment of the rabbits was not higher than 0.6°C, and the total temperature increment of the three rabbits was not higher than 1.3°C (refer to Table 1), indicating that BSMs are not pyrogenic and that they adhere to thermal test requirements for medical materials.

### 3.2. Cytotoxicity of BSMs

**Figure 2:**
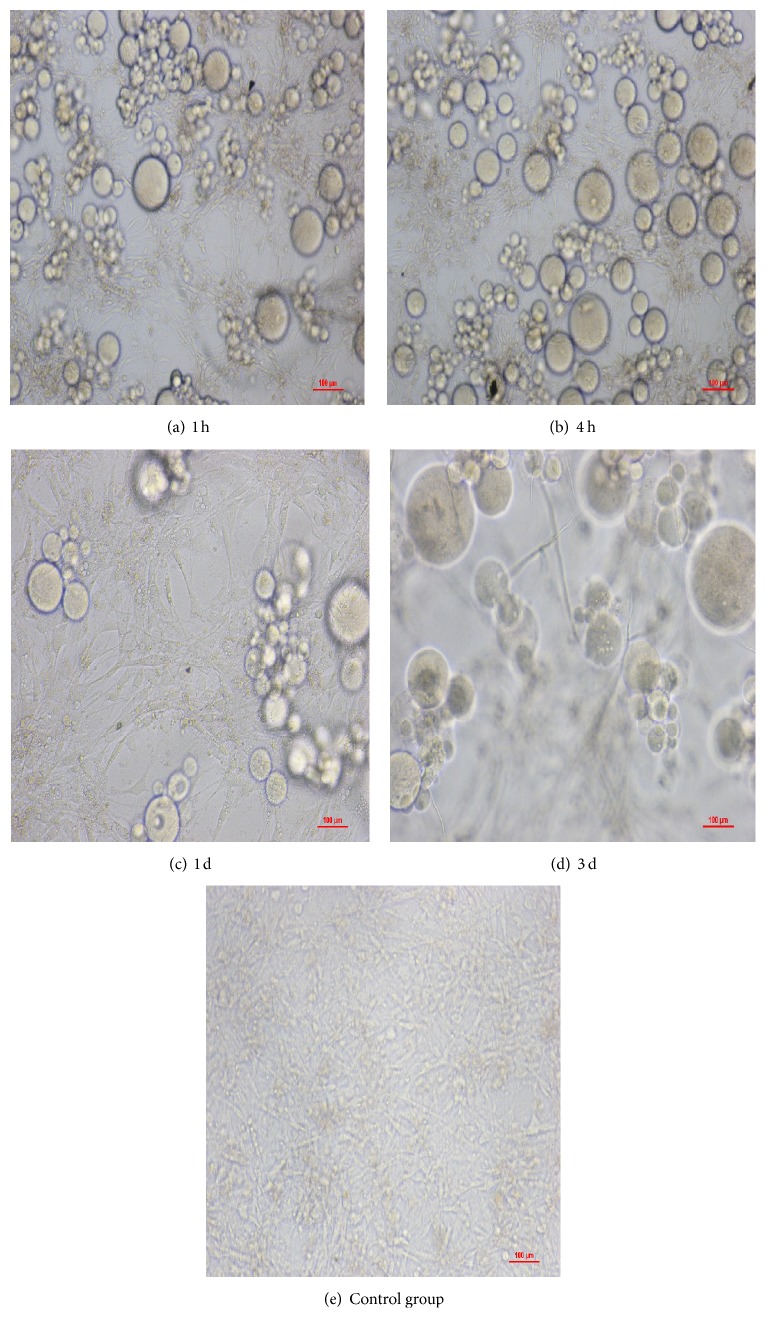
Mouse fibroblasts growth condition after culture of BSMs and mouse fibroblasts. Mouse fibroblasts grew well within the first hour and also the fourth hour while the growth was worse than the control group and it was not different from the control group at the first day, but growth of the two cells stopped with BSMs at the third day; mouse fibroblasts grew well in the control group.

**Figure 3 fig3:**
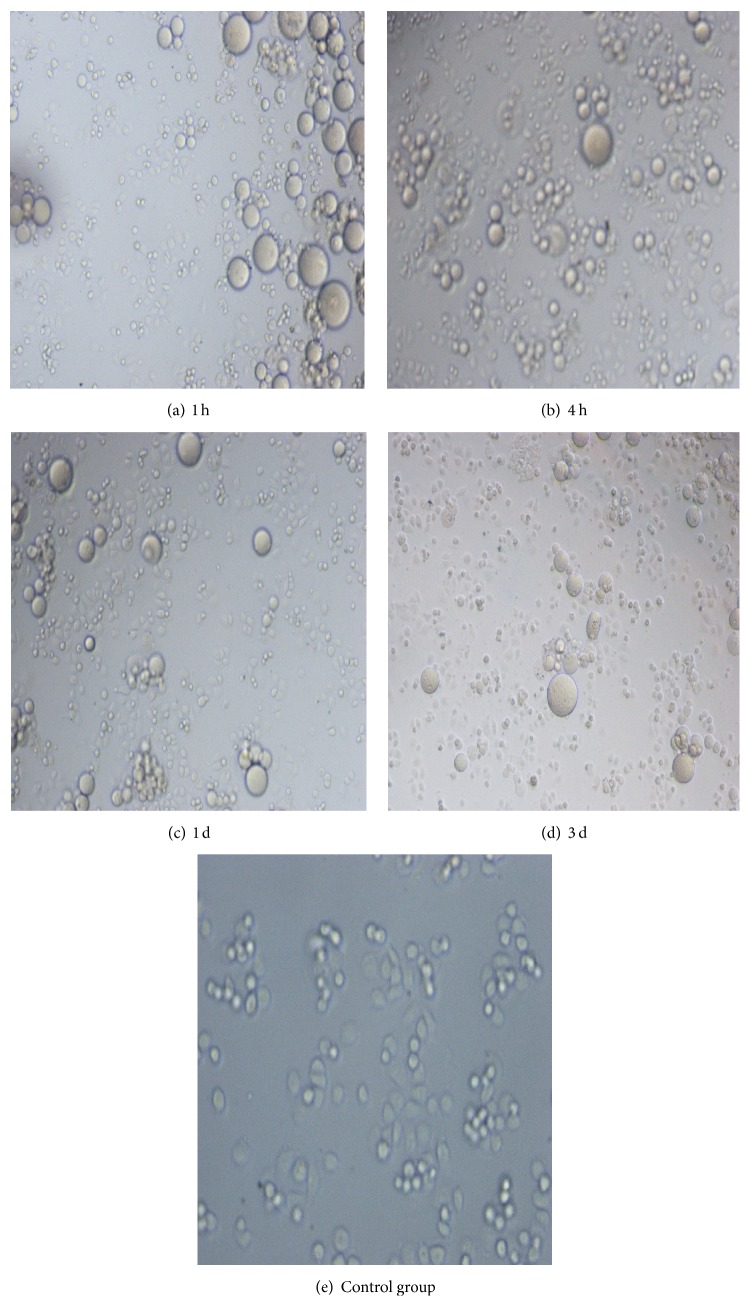
Human umbilical vein endothelial cells growth condition after culture of BSMs and human umbilical vein endothelial cells. Human umbilical vein endothelial cells grew well within the first hour and also the fourth hour while the growth was worse than the control group and it was not different from the control group at the first day, but growth of the two cells stopped with BSMs at the third day; human umbilical vein endothelial cells grew well in the control group.

**Figure 4:**
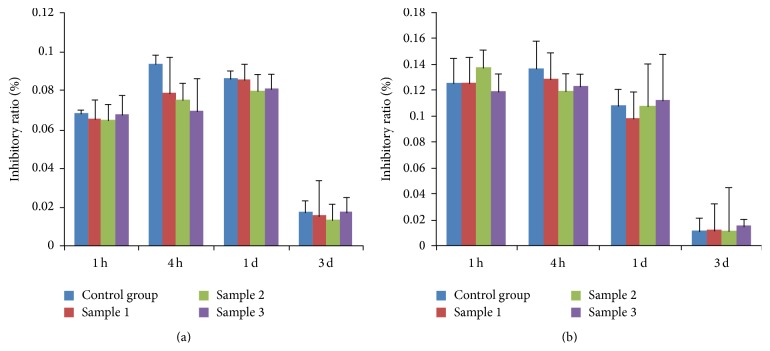
(a) BSM growth inhibitory ratio of mouse fibroblasts. (b) BSM growth inhibitory ratio of human umbilical vein endothelial cells.

Two normal cell lines (Figures 2 [Fig fig3]–4), mouse fibroblasts and human umbilical vein endothelial cells, were exposed to BSMs for 1 h, 4 h, and 24 h, and the cytotoxicity of BSMs was determined using the MTT assay. Mouse fibroblasts and human umbilical vein endothelial cells grew well at the first hour and also the fourth hour while the growth was worse than the control group and it was not different from the control group at the first day, but growth of the two cells stopped with BSMs at the third day; proliferation ratio of the two cells is the same without statistical difference as that in the control group at the same time of the first three hours. No toxicity was observed for any of the incubation periods. Three days later, the two cell lines had almost stopped growing.

### 3.3. Blood Compatibility of BSMs

**Figure 5:**
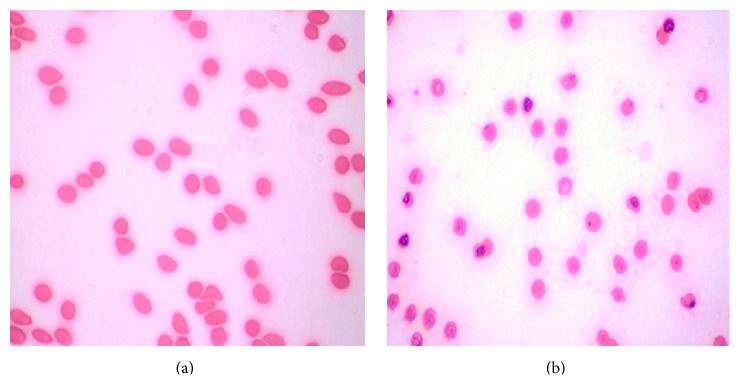
Erythrocyte observed with light microscope after Wrights staining. Rabbit erythrocytes were treated with BSMs for 24 h. The red blood cells were all sunk down and the supernatant was colorless and transparent. Consequently, no erythrocyte lysis was observed, and no abnormalities of red blood cell morphology were observed by light microscopy after Wright staining.

**Figure 6:**
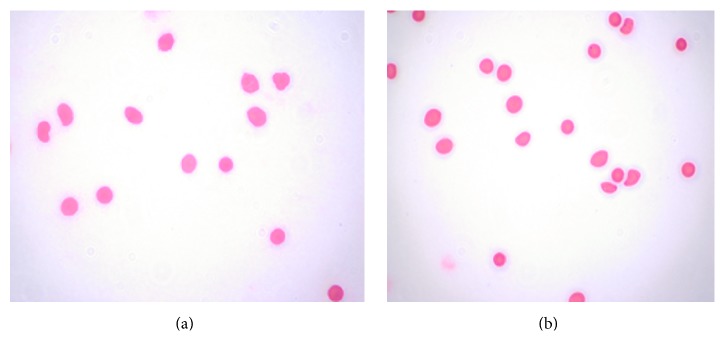
Erythrocyte observed with light microscope after Wrights staining. Erythrocyte state got no obvious abnormality under observation with light microscope after Wrights staining.

Rabbit erythrocytes were treated with BSMs for 24 h. The red blood cells were all sunk down and the supernatant was colorless and transparent. Consequently, no erythrocyte lysis was observed, and no abnormalities of red blood cell morphology were observed by light microscopy after Wright staining (Figure 5). Erythrocyte state got no obvious abnormality under observation with light microscope after Wrights staining (Figure 6).

### 3.4. Intramuscular Implantation of BSMs

**Figure 7:**
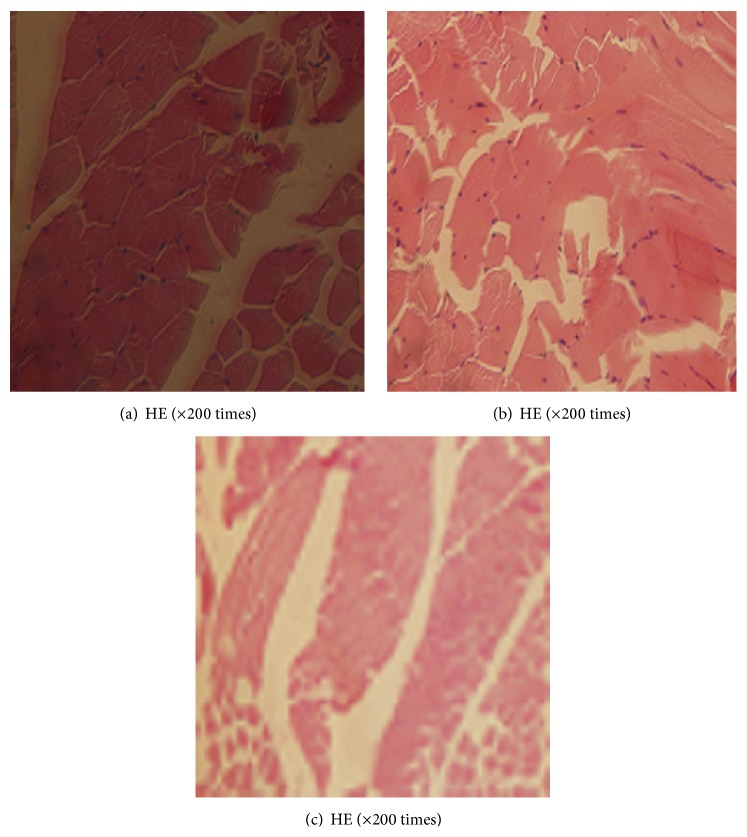
((a)–(c)) Pathological results of muscle tissue at the implantation site at 1, 4, and 8 weeks.

Experimental rabbits observed grossly were free from deterioration, exudation, formation of granulation tissue, adhesion of surrounding muscle tissue, and significant differences at both sides in the part planted at the first, fourth, and eighth week. The implant sites were free from necrosis and deterioration after muscle implantation during the study period. As shown in Figures 7(a)–7(c), the inflammatory reaction was gradually lessened and finally disappeared after a prolonged time.

### 3.5. BSM Embolization of the Renal Artery

All of the experimental rabbits presented normally after embolization and during the observation period. As shown in Table 2, BUN and Cr levels rose clearly on the third day after the surgery in the experimental and control groups, most clearly on the seventh day, and recovered to normal at the fourteenth day. ALT and AST levels were elevated on the third day after embolization and recovered to normal levels on the seventh day. Differences between the AST, ALT, BUN, and Cr levels of the two groups showed no significant difference (*P* > 0.05) during the corresponding period.

**Figure 8:**
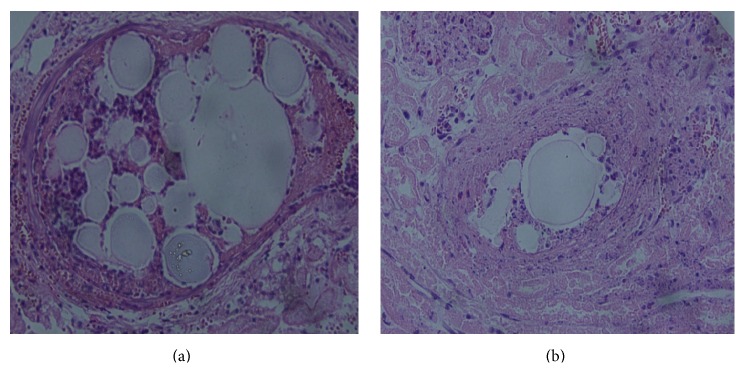
(a) Vascular Masson staining indicated a light-blue, complete vascular wall without rupture and damage at the fourth week after BSM embolization in the right renal artery; (b) vascular intima broke at the fourth week after PVA granule embolization in the right renal artery.

The inflammatory response concerning the renal artery of the two groups was similar, mainly for the exudation of neutrophilic granulocytes at the early stage and then for the exudation of lymphocytes and neutrophilic granulocytes during the first week. PVA granule vascular injury was not obvious during the second week, was aggravated during the fourth week, and was alleviated after the sixth week; however, BSM vascular injury was not very obvious (Figures 8(a) and 8(b)).

## 4. Discussion

The biocompatibility of medical material arises from complex biological, physical, and chemical reactions after the mutual effect of medical biological materials and the human body, primarily including blood compatibility and tissue compatibility [8]. Blood compatibility refers to results from the mutual effect of medical materials and organism blood, while tissue compatibility refers to results from the mutual effect of medical materials and organism tissue. Currently, there are two primary methods to evaluate material biocompatibility [9]. The first test is performed in vitro to observe the impact of the medical material or medical material solution on the growth, metabolism, and proliferation of tissue cells. Second, the tissue compatibility of the medical material is evaluated in vivo. Evaluation and research on the blood compatibility of biological materials are very important strategies [10] and consist of testing in vivo to evaluate the tissue compatibility of a medical material. Material blood compatibility is evaluated through erythrocyte dissolution by contact with the material in vitro using the hemolysis test. Rabbit erythrocyte toxicity [11] is reflected sensitively, so the hemolysis test is an important method for evaluating the blood compatibility of medical materials. Generally speaking, the material is toxic if hemolysis occurs or if erythrocytes appear abnormal when the biological materials are in contact with blood [12]. This experiment utilized erythrocytes from New Zealand rabbits and BSMs, and the negative control and positive control were free from erythrocyte lysis. The erythrocytes showed no obvious abnormality under observation with a microscope after Wright staining, indicating that BSMs did not damage erythrocytes and possessed good blood compatibility.

Pyrogen is the general term of substances that can make homeothermic temperature rise abnormally in a microorganism compound consisting of phospholipid, lipopolysaccharide, and protein [13]. This experiment adopted the thermal test in New Zealand rabbits, and according to Chinese Pharmacopoeia, rabbits have the same reaction to pyrogens as humans. Experimental rabbits were selected according to experimental requirements and were maintained in an environment at the same temperature, kept quiet, and avoided strong light and noise as well as any factors inducing animal restlessness one or two days before the thermal test. All rabbits were healthy and qualified, and female rabbits were not pregnant. In addition, all rabbits were fed with the same forage and maintained the same weight with normal activity appetite and drainage seven days before the test. A thermometer with ±0.1°C precision was used to measure the temperature of Japanese white rabbits. The placement depth of the rectal thermometer and the plugging time were the same in each rabbit (6 cm deep and 1.5 min). Temperature was measured once every 30 min, generally twice total; the difference between the two temperatures measured should not have been more than 0.2°C. Then, the normal temperature of the rabbit was obtained from the average value of the two temperatures measured. None of the experimental rabbits were used in the group if one rabbit was found to meet the exclusion criteria. The temperature change of the three J rabbits should be in accordance with standards of the thermal test in Chinese Pharmacopoeia and free from pyrogen generation.

Currently, cell toxicity tests significantly affect biocompatibility evaluations [14]. Cells may be damaged initially by the toxic components of medical materials through cell biochemical reactions and biological molecular structure changes as well as by metabolism and functional changes. Because those changes cannot be detected by morphological methods, we must consider their impact on cell growth and the adhesion, proliferation, and metabolism of the materials and treat surviving functional cells and cell growth and proliferation conditions as indications of the biocompatibility of medical materials [15, 16]. Presently, there are two primary methods to evaluate the cell toxicity of biological materials: the direct plantation method in vivo and compound cell culture method in vitro [17]. The direct plantation method in vivo involves planting medical materials in vivo and removing them at different times for gross pathology and histological examination. The mixed cell cultivation method in vitro involves the culture of medical materials in vitro with various cells to examine the effect on the growth and proliferation of the cells [18, 19]. The present study adopted mixed cultivation of BSMs and mouse fibroblasts and human umbilical vein endothelial cells in vitro to observe the impact of BSMs on the growth and proliferation of the two cell types. Mouse fibroblasts and human umbilical vein endothelial cells grew well during the first hour as well as during the fourth hour, but the growth was worse than that of the control group, which may indicate an adaptation process of the cells. Additionally, there was no difference from the control group on the first day, but the two cell types treated with BSMs stopped growing on the third day because of a nutritional shortage for the cells. In contrast to the control group, the proliferation ratio of the two cell types was nearly similar at the same time of the previous three periods. No significant difference indicated the lack of toxicity of BSMs to the cell types.

The pathology and healing response of organisms to implantation is an important component in the evaluation of the biocompatibility of BSMs and in the detection of cell toxicity [20]. Wound healing is the response of tissue and cells to implantation, and BSM infusion can cause this effect in organisms. Although the degree of inflammation is related to the compatibility between medical materials and implanted surrounding tissues, inflammation is minimal if the tissue possesses good compatibility with the surrounding tissues. It has been reported [21–23] that the tissue response to BSM injection includes acute and chronic inflammation. Lobocytes, lymphocytes, erythrocytes, and monocytes can be found at the early stage. Chronic inflammatory cells mainly consist of monocytes and microphagocytes, and the tissue response mainly results from accelerated degradation after the engulfment of microphagocytes. BSMs are considered foreign matter after they are implanted in the animal body, leading to an inflammatory response in the organism and the agglomeration of inflammatory cells and the release of various inflammatory factors. The inflammation results in fibroblast proliferation, and the foreign matter is surrounded by large amounts of collagen fiber if the inflammation is finally controlled. The test result indicates that Japanese white rabbits are free from deterioration, exudation, formation of granulation tissue, adhesion of surrounding muscle tissue, and significant differences at both sides in the region implanted at the first, fourth, and eighth weeks. Histopathology detection shows minimal inflammation of the cells, indicating that BSMs possess good tissue compatibility.

The vascular inflammatory response and organ functional change of the target organ to the embolization material for interventional therapy are important indexes to evaluate the compatibility of the material and organ [24]. A strong inflammatory response of the embolized vessel or great functional damage of the target organ indicates that the material has poor compatibility with the organ and a substantial toxic tissue response [25]. After BSM renal artery embolization, BUN and Cr levels rose on the third day after surgery in the experimental and control groups, most obviously on the seventh day, and recovered to normal on the fourteenth day. The ALT and AST levels rose on the third day after embolization and recovered to normal on the seventh day. The differences in the AST, ALT, BUN, and Cr levels between the two groups did not display any statistical differences during the corresponding period. Measuring liver and renal enzymes after arterial embolization provides key information about the general state of organ function. It is common clinically for liver and renal enzymes to increase transiently following arterial embolization, peaking at 24 to 36 hours before returning to baseline after 5 to 7 days. As expected, after embolizing with BSMs, we found all enzymes to increase transiently, reaching a peak and returning to baseline. Taken together, these results showed that embolization therapy caused a minimal transient elevation of enzymes, without significant compromise of liver and kidney functions. The pathological results indicated that the inflammatory response around the renal artery of the two groups was similar, primarily regarding the exudation of neutrophilic granulocytes at the early stage and subsequently for the exudation of lymphocytes and neutrophilic granulocytes after the first week. BSMs had no direct toxic effect on the liver, kidney, and renal vessel compared with PVA. The temporary dysfunction of the liver and kidney that resulted from embolization recovered to normal after one or two weeks.

## 5. Conclusions

BSMs caused neither hemolysis nor cytotoxicity. The results of the implantation test and transcatheter renal embolization indicated that BSMs had good tissue biocompatibility and no adverse effects, such as hepatotoxicity and renal toxicity, indicating their promising potential for use as a highly biocompatible embolic agent.

## Figures and Tables

**Table 1:** Rectal temperature of experimental rabbits before and after BSMs injection (°C).

Rabbit/time	Before injection	30 min	60 min	90 min	120 min	150 min	180 min	Temperature increment
Rabbit 1	38.5	38.9	39.0	38.8	38.8	38.7	38.9	0.4
Rabbit 2	38.6	38.9	38.9	38.9	38.8	38.8	38.7	0.3
Rabbit 3	38.5	39.0	38.8	38.9	38.8	38.7	38.6	0.4
Total								1.1
Temperature increment								

**Table 2:** Renal and liver function at different times.

Group	Time	ALT (U/L)	AST (U/L)	BUN (*µ*mol/L)	Cr (mmol/L)
Experimental group	0 d	33.1 ± 12.1	29.8 ± 9.6	6.2 ± 2.1	59.2 ± 14.3
	1 d	28.3 ± 11.5	37.3 ± 13.2	5.9 ± 2.6	58.3 ± 24.1
	3 d	72.1 ± 25.6	63.7 ± 19.5	21.5 ± 7.6	140.6 ± 24.5
	7 d	38.4 ± 11.6	31.5 ± 7.9	19.2 ± 4.3	161.1 ± 36.2
	14 d	37.5 ± 5.9	30.3 ± 6.2	6.1 ± 1.9	96.4 ± 28.4
	21 d	31.5 ± 6.3	30.8 ± 7.3	7.3 ± 1.9	65.2 ± 13.5
	4 w	28.1 ± 7.5	32.6 ± 9.8	6.4 ± 2.1	63.9 ± 17.2
	6 w	26.6 ± 9.2	31.5 ± 10.5	6.7 ± 2.1	67.3 ± 15.7
	8 w	27.3 ± 9.3	30.5 ± 16.2	5.7 ± 1.8	64.8 ± 11.8
	12 w	36.7 ± 7.4	35.3 ± 9.3	6.5 ± 1.4	71.3 ± 17.6

Control group	0 d	35.7 ± 13.3	25.6 ± 9.2	5.3 ± 1.7	67.3 ± 16.4
	1 d	29.4 ± 14.6	35.2 ± 11.7	6.7 ± 2.4	73.1 ± 23.5
	3 d	71.1 ± 16.6	67.2 ± 21.6	19.3 ± 7.4	146.2 ± 32.7
	7 d	40.2 ± 12.1	29.4 ± 7.9	16.2 ± 3.5	135.9 ± 15.8
	14 d	36.1 ± 11.7	26.7 ± 9.3	6.4 ± 1.7	85.4 ± 21.5
	21 d	34.8 ± 13.3	32.6 ± 7.6	6.2 ± 1.9	63.1 ± 19.4
	4 w	32.9 ± 8.4	35.7 ± 9.8	5.5 ± 1.7	68.4 ± 13.1
	6 w	26.8 ± 3.7	26.2 ± 5.3	4.7 ± 1.6	61.2 ± 11.6
	8 w	29.4 ± 9.5	29.8 ± 5.1	5.1 ± 1.1	66.4 ± 12.4
	12 w	29.7 ± 8.2	31.3 ± 6.5	5.9 ± 1.7	70.3 ± 19.3
